# A Comparison of the Quality of Life of People With Epilepsy Receiving Home-Based and Clinic-Based Epilepsy Care Using the European Quality of Life Five-Dimension Three-Level (EQ-5D-3L) Scale

**DOI:** 10.7759/cureus.35045

**Published:** 2023-02-16

**Authors:** Shivani Kalra, Triza Jiwan, Gagandeep Singh, Parshotam L Gautam, Amit Bansal

**Affiliations:** 1 Critical Care, Shaheed Kartar Singh Sarabha (SKSS) College of Nursing, Ludhiana, IND; 2 Psychiatry and Nursing, Dayanand Medical College and Hospital, Ludhiana, IND; 3 Neurology, Dayanand Medical College and Hospital, Ludhiana, IND; 4 Critical Care Medicine, Dayanand Medical College and Hospital, Ludhiana, IND; 5 Gastrointestinal (GI) and Liver Sciences, Satguru Partap Singh (SPS) Hospitals, Ludhiana, IND

**Keywords:** epilepsy, quality of life (qol), neurological disorder, seizures, people with epilepsy, eq-5d-3l

## Abstract

Background and objective

Epilepsy is a chronic neurological condition that, both physically and psychologically, puts a person at risk for poor quality of life (QOL). People with epilepsy (PWE) may experience shame, fear, and rejection and feel discriminated against, hence avoiding social interactions. To avoid being labeled as having epilepsy, patients may conceal their disease and refuse medical attention, which can lead to treatment discontinuation and significantly impact the quality of life. Epilepsy care in India has fallen back on primary care physicians because there are not enough neurologists available to treat the condition. Home-based care (HBC) may overcome many barriers by providing free antiepileptic drugs (AEDs), eliminating the “distance to a health facility,” and providing correct information that may improve QOL. This study is therefore conducted to compare the QOL between people with epilepsy receiving home-based care (HBC) and routine clinic-based care (CBC).

Methodology

The people with epilepsy enrolled in this study were already part of a community-based randomized controlled trial conducted to compare the effect of regular home-based epilepsy care with routine clinic-based epilepsy care on antiepileptic adherence among urban and peri-urban areas of Ludhiana, Punjab, India (explained further in the study). The present study is a cohort study where the two cohorts, one receiving home-based epilepsy care (n = 97) and the other receiving routine clinic-based epilepsy care (n = 76), were compared for QOL at two points in time, i.e., at baseline (at enrolment) and after 24 months of receiving epilepsy care, using the European Quality of Life Five-Dimension Three-Level (EQ-5D-3L) scale.

Results

The mean EQ-5D-3L index scores for the HBC group at baseline were 0.88 ± 0.15, and after 24 months, the scores increased to 0.94 ± 0.17. The baseline mean index scores for the CBC group were 0.89 ± 0.21, and after 24 months, the value increased to 0.90 ± 0.19. The mean difference in QOL in the HBC group showed a higher difference than in the CBC group (0.06 ± 0.1 versus 0.01 ± 0.1), but the difference was found to be statistically not significant (p = 0.067). As per the five dimensions of the EQ-5D-3L scale, i.e., mobility, self-care, usual activities, pain/discomfort, and anxiety/depression, there was a decrease in the number of PWE reporting problems among both groups after 24 months of epilepsy care. Sociodemographic and clinical variables such as level of education, working status, age at the onset of seizures, frequency of seizures, treatment regimen, presence of comorbidities, and adverse drug reactions significantly affect the QOL of people with epilepsy at p < 0.05.

Conclusion

The results of the study emphasize that epilepsy has a negative impact on QOL. The results showed a higher QOL among the people in the HBC group as compared to the CBC group, but the difference was not statistically significant. There was an improvement in QOL from baseline after dedicated care in both groups. The problems related to mobility, self-care, usual activities, pain/discomfort, and anxiety/depression have been significantly reduced in the HBC group. Having low levels of education, not having a job, starting to have seizures at a young age, having seizures more often, receiving more than one type of treatment, and the presence of other health problems and side effects are factors associated with poor QOL among people with epilepsy.

## Introduction

Epilepsy is a chronic neurological disorder characterized by recurrent seizures, which may vary from a brief lapse of attention or muscle jerks to severe and prolonged convulsions. The seizures are caused by sudden, usually brief disruptions of electrical activity in the brain cells [1,2]. The International League Against Epilepsy (ILAE) defined epilepsy as a disease diagnosed by any of the following criteria: (i) at least two unprovoked (or reflex) seizures occurring more than 24 hours apart, (ii) one unprovoked (or reflex) seizure and a probability of further seizures similar to the general recurrence risk (at least 60% recurrence risk) after two unprovoked seizures occurring over the next 10 years, or (iii) having an epilepsy syndrome [3]. According to the World Health Organization (WHO), approximately 50 million people worldwide suffer from epilepsy, with more than 85% living in developing countries. Based on a conservative estimate of 1% as the prevalence of epilepsy, India has more than 12 million PWE, accounting for nearly one-sixth of the global burden [4,5].

Epilepsy affects patients’ physical, psychological, social, cognitive, and behavioral health and puts a person at risk for poor health-related quality of life (HRQOL). The psychological impact is related to the uncertainty of seizures, fear of recurrent seizures, associated physical injuries, treatment-related side effects, lifestyle restrictions, physical difficulties, and perceived stigmatization. As a result of stigma, people with epilepsy (PWE) may experience shame, fear, and rejection and feel discriminated against, hence avoiding social interactions. To avoid being labeled as having epilepsy, patients may conceal their disease and refuse medical attention.

Additionally, the availability of epilepsy care in low- and middle-income countries (LMICs) is problematic due to both supply-side and demand-side constraints (e.g., lack of antiepileptic drugs (AEDs), distances to healthcare facilities, a lack of expertise in treating epilepsy, treatment costs, cultural beliefs regarding epilepsy, and faith in traditional treatment providers). Together, these factors have the potential to result in psychological stress and low self-esteem, which can lead to discontinuation (nonadherence) of treatment. Nonadherence results in seizure relapse, status epilepticus, hospitalizations, increased healthcare costs, and, in rare cases, sudden unexpected death from epilepsy (SUDEP) and could significantly impact the quality of life (QOL). The QOL is also influenced by the type of seizures, frequency of seizure, treatment regimen, duration of epilepsy, presence of comorbidities, and adverse effects, and by some sociodemographic characteristics such as age, marital status, and employment status. Therefore, appropriate care must be provided to prevent complications related to epilepsy [6-8].

Epilepsy care in India has fallen back on primary care physicians because there are not enough neurologists available to treat the condition. The WHO encourages primary healthcare providers to deliver epilepsy care in countries and regions where adequate facilities are not available [9]. Home-based epilepsy care (HBC) may overcome barriers by providing routine, free AEDs, eliminating the “distance to a health facility,” and providing information and advice to reduce stigma and false beliefs, as well as supporting self-management by primary care workers. Therefore, a community-based cluster randomized trial named Community Interventions for Epilepsy (CIFE) was conducted to ascertain whether home-based care with the community and primary healthcare workers’ support improves adherence to AEDs over routine clinic-based care (CBC) among people with epilepsy [10,11]. The present study was an extension of this community-based trial, where the same groups receiving home-based epilepsy care and routine clinic-based epilepsy care were followed to assess and compare the quality of life.

Over the past few decades, many quality of life questionnaires have been developed and used to assess the quality of life of patients with various diseases. Some are disease-specific, and others are generic. While disease-specific instruments are more sensitive to detecting changes in health related to disease, generic instruments were built to assess and compare the lifestyles of people suffering from various pathologies [12-14]. The European Quality of Life Five-Dimension Three-Level (EQ-5D-3L) scale, which is a generic questionnaire, was used in this study with the aim to compare QOL among people with epilepsy receiving home-based epilepsy care and clinic-based epilepsy care.

## Materials and methods

**Figure 1:**
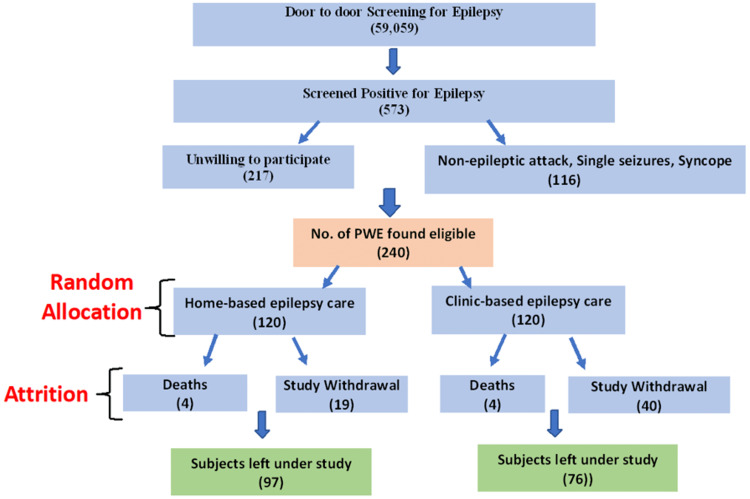
Flowchart showing PWE selection PWE: people with epilepsy

This is a cohort study in which the quality of life of two cohorts receiving home-based and routine clinic-based epilepsy care was compared. The study included a total of 173 PWE, more than one year of age, screened, diagnosed, and recruited by a panel of neurologists under a community-based trial (the Community Interventions for Epilepsy (CIFE) project) [10,11]. PWE were assessed monthly for 24 months by trained field-workers and neurologists. CIFE was a two-step cluster-randomized trial during which 59,509 people were screened for epilepsy in urban and peri-urban areas of Ludhiana, Punjab. The trained field-workers carried out door-to-door screening among 24 selected clusters of around 2,000 people each using a validated questionnaire. Screen-positive people were then invited for evaluation by neurologists specializing in epilepsy at a tertiary care hospital facility. It was finally conducted on 240 people with epilepsy, equally divided among both groups, to compare the effect of regular home-based care with the routine clinic-based care provided by neurologists on AED adherence in people with epilepsy (Figure 1).

PWE under the routine clinic-based epilepsy care were asked to attend monthly clinics at the Government District Hospital for review visits and to receive free antiepileptic drugs (AEDs), while those under the home-based epilepsy care received an interventional package comprising (a) free delivery of AEDs, (b) education and counseling about self-management, social functioning, and stigma abrogation, and (c) adherence monitoring, all provided at home on a monthly basis by study personnel with qualifications equivalent to auxiliary nurse midwives (ANMs). The duration of the trial was from December 2017 to August 2020 (24 months for each patient).

In this present study, the same cohort of PWE receiving home-based and routine clinic-based epilepsy care were assessed for QOL using the EQ-5D-3L scale. All PWE who have completed 24 assessments (over 24 months) under the CIFE project, are older than one year, and are willing to participate in the study were selected as part of the study using the total enumerative sampling technique. The study was approved by the Research and Development Centre of Dayanand Medical College and Hospital (approval number DMCH/R&D/2018/825). Informed consent was obtained from all subjects over the age of 18, as well as from a parent or guardian in the case of children (in addition to oral consent from children 7-11 years old and written consent from children over 12 years old). The details of clinical condition, frequency of seizures, treatment regimen, presence of comorbidities, and others were taken from medical records. The selected PWE were interviewed for demographic data, clinical profile, and quality of life using the EQ-5D-3L scale.

The EQ-5D-3L scale is a generic instrument that assesses the overall condition of the patient (physical, psychological, and social) regardless of the pathology [15]. It helps compare the lifestyles of groups of subjects to many pathologies. The questionnaire has two parts: the EQ-5D descriptive system and the EQ visual analog scale (EQ-VAS).

EQ-5D descriptive system

This includes five dimensions of health: mobility, self-care, usual activities, pain or discomfort, and anxiety or depression. Each dimension has three levels: no problems, some problems, and extreme problems. Patients were asked to select one of three health levels for each of the five dimensions. The answers given can be combined into a number of five digits that describes the patient’s health state, where “11111” is a perfect health state. The results of the health state can be transformed into a single index value using the value sets given for different countries. As the value sets are not available for India, the value set for China has been used for the study (as per the EQ-5D-3L instrument guide instructions).

EQ visual analog scale

The EQ-VAS records the patient’s self-rated health on a vertical visual analog scale where the endpoints are labeled “best imaginable health state” and “worst imaginable health state.” The VAS can be used as a quantitative measure of health outcomes that reflect the patient’s own judgment. The people were then classified based on their self-reported health states as very good (81-100), good (51-80), normal (31-50), and bad (0-30).

The EuroQOL International Group provided permission to use the instrument via email. The scale was already available in Hindi and Punjabi on the EuroQOL website. The questionnaire was validated by the experts for English, Hindi, and Punjabi, and the reliability of the scale was tested using Cronbach's alpha (EQ-5D descriptive system: r = 0.94; EQ-VAS: r = 0.98). Data were collected through face-to-face interviews with the researchers. The questionnaire was pretested on a selected group of people with epilepsy to ensure that all the questions were properly understood by the respondents. Changes were made, and the final version of the questionnaire was used to collect the data. The groups were then compared for quality of life at baseline and after 24 months of receiving home-based and routine clinic-based epilepsy care. The Statistical Package for the Social Sciences (SPSS) version 20 (IBM SPSS Statistics, Armonk, NY, USA) was used to analyze the data. The results were expressed as means and standard deviation (SD) for variables that met the criteria of normality. For variables that were not normally distributed, non-parametric tests (Mann-Whitney U and Kruskal-Wallis tests) were used to find out the association of EQ-5D scores with sociodemographic and clinical variables. The statistical significance level was set at 0.05.

## Results

Sociodemographic profile of people with epilepsy

As per the sociodemographic profile of people with epilepsy, the majority were in the age group of 13-30 years, i.e., 87 (50.2%), with a mean age of 25.64 ± 15.1. Most of the PWE were male (65.3%) and unmarried (61.8%). Fifty-four (31.2%) PWE were secondary or senior secondary educated. Only about one-third of people with epilepsy (37.6%) were working, and of them, almost half were unskilled workers (40.1%). There was no difference between the groups as checked using the chi-square test of homogeneity (Table 1).

**Table 1:** Sociodemographic profile of PWE among the two groups (N = 173) Mean age: 25.64 ± 15.1 PWE: people with epilepsy, HBC: home-based care, CBC: clinic-based care, NS: not significant

Sociodemographic variables	Groups		Total	ᵡ^2^ value	p value
	HBC (n = 97)	CBC (n = 76)			
	Number (%)	Number (%)			
Age (in years)					
13-30	46 (47.4)	41 (54)	87 (50.2)	2.25	0.521^NS^
30-50	36 (37.1)	25 (32.9)	61 (35.3)		
50-70	15 (15.5)	9 (11.8)	24 (13.9)		
70-90	-	1 (1.3)	1 (0.6)		
Gender					
Male	65 (67.1)	48 (63.1)	113 (65.3)	0.279	0.597^NS^
Female	32 (32.9)	28 (36.8)	60 (34.7)		
Marital status					
Unmarried	59 (60.7)	48 (63.2)	107 (61.8)	1.622	0.654^NS^
Married	35 (36.1)	23 (30.3)	58 (33.5)		
Widow/widower/separated	2 (2.1)	3 (3.9)	5 (2.9)		
Divorced	1 (1.1)	2 (2.6)	3 (1.7)		
Education					
Illiterate	19 (19.6)	27 (35.5)	46 (26.6)	10.21	0.337^NS^
Primary	20 (20.6)	12 (15.8)	32 (18.5)		
Secondary/senior secondary	38 (39.2)	16 (21.1)	54 (31.2)		
Graduate and above	4 (4.1)	3 (3.9)	7 (4.1)		
Studying	16 (16.5)	18 (23.7)	34 (19.7)		
Working status					
Working	38 (39.2)	27 (35.5)	65 (37.6)	0.242	0.623^NS^
Not working	59 (60.8)	49 (64.5)	108 (62.4)		
Occupation of those who are working (HBC: n = 38; CBC: n = 27)					
Professional	2 (5.3)	-	2 (3.1)	4.058	0.398^NS^
Semiskilled worker	13 (34.2)	6 (22.2)	19 (29.2)		
Skilled worker	11 (28.9)	7 (25.9)	18 (27.6)		
Unskilled worker	12 (31.5)	14 (51.8)	26 (40.1)		

Clinical profile of people with epilepsy

As per the clinical profile, the majority of people with epilepsy were diagnosed with symptomatic focal epilepsy in both home-based care and clinic-based care, i.e., 40 (41.2%) and 32 (42.1%), respectively. Among the home-based care group, the age of onset of seizures was less than five years among most PWE (34.1%), while the duration of seizures was 10-15 years in the majority of them. The majority of PWE in the clinic-based care group had seizures between the ages of 10 and 15, and the duration was five years. The majority of PWE in both the HBC and CBC groups had monthly seizures, i.e., 28.7% and 35.5%, respectively. Most of the PWE in the HBC group were prescribed polytherapy (54.6%) as a treatment regimen, while in the CBC group, an equal number of PWE were prescribed monotherapy and polytherapy, i.e., 50%. Adverse drug reactions were present in 26 (26.8%) PWE in the home-based care group as compared to 13 (17.1%) in the clinic-based care group. Comorbidities were present in approximately half of the PWE among both groups, i.e., 47 (48.5%) and 31 (40.8%), respectively. After 24 months, 27 (27.8%) PWE had controlled epilepsy, 24 (24.7%) had drug-resistant epilepsy (DRE), and 46 (47.4%) had indeterminate epilepsy (which could not be classified under the above two categories). As depicted by the p value, there was no significant difference between the groups as per their clinical profile (Table 2).

**Table 2:** Clinical profile of PWE receiving home-based and clinic-based epilepsy care (N = 173) PWE: people with epilepsy, HBC: home-based care, CBC: clinic-based care, NS: not significant

Clinical profile	Groups		ᵡ^2^ value	p value
	HBC (n = 97)	CBC (n = 76)		
	Number (%)	Number (%)		
Diagnosis				
Idiopathic focal epilepsy	2 (2.1)	1 (1.3)	3.145	0.790^NS^
Idiopathic generalized epilepsy	18 (18.6)	10 (13.1)		
Symptomatic focal epilepsy	40 (41.2)	32 (42.1)		
Symptomatic generalized epilepsy	5 (5.2)	6 (7.9)		
Unestablished	32 (33.1)	27 (35.5)		
Age at onset of seizures (in years)				
<5	33 (34.1)	18 (23.7)	7.865	0.164^NS^
5-10	16 (16.5)	10 (13.1)		
10-15	11 (11.3)	20 (26.3)		
15-20	11 (11.3)	11 (14.5)		
20-25	15 (15.5)	10 (13.1)		
>25	11 (11.3)	7 (9.2)		
Duration of epilepsy (in years)				
<5	19 (19.6)	22 (28.9)	6.173	0.290^NS^
5-10	21 (21.6)	12 (15.8)		
10-15	22 (22.6)	8 (10.5)		
15-20	13 (13.4)	9 (11.8)		
20-25	7 (7.2)	4 (5.3)		
>25	15 (15.5)	21 (27.6)		
Frequency of seizures				
Daily	10 (10.3)	4 (5.3)	5.747	0.332^NS^
Weekly	9 (9.3)	8 (10.5)		
Monthly	28 (28.7)	27 (35.5)		
Annually	6 (6.2)	4 (5.3)		
Sporadic	32 (33.1)	17 (22.4)		
Biannually	12 (12.4)	16 (21.1)		
Treatment regimen				
Monotherapy	44 (45.4)	38 (50)	2.134	0.544^NS^
Polytherapy	53 (54.6)	38 (50)		
Adverse drug reactions				
Present	26 (26.8)	13 (17.1)	2.296	0.130^NS^
Not present	71 (73.2)	63 (82.9)		
Comorbidities				
Present	47 (48.5)	31 (40.8)	1.011	0.315^NS^
Not present	50 (51.5)	45 (59.2)		
Seizure control				
Controlled epilepsy	27 (27.8)	23 (30.3)	2.838	0.242^NS^
Drug-resistant epilepsy	24 (24.7)	26 (34.2)		
Indeterminate	46 (47.4)	27 (35.5)		

Assessment of self-reported quality of life using the EQ-5D-3L scale

**Figure 2:**
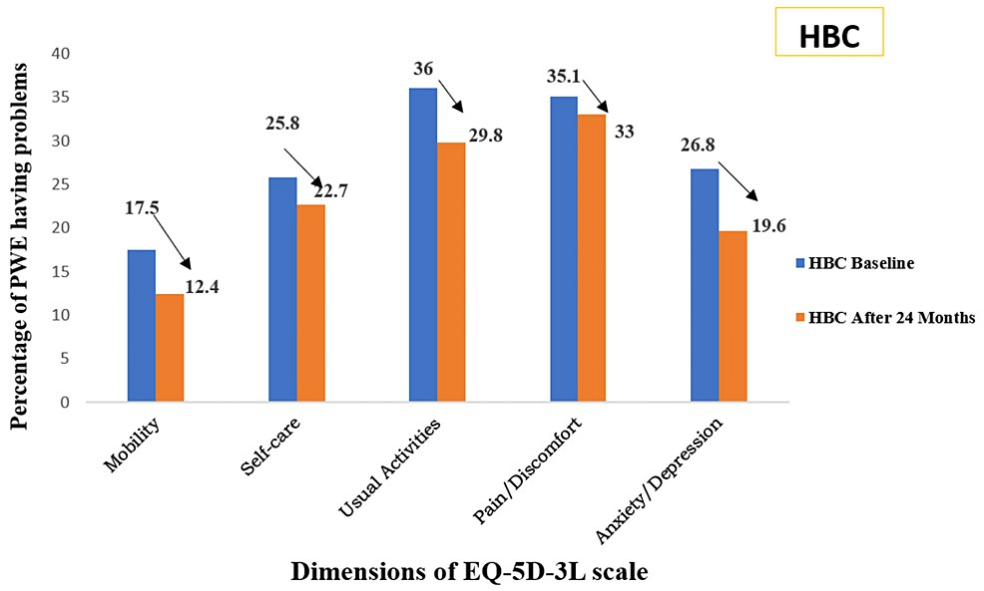
Percentage of PWE having some problems (level 2 + 3) at baseline and after 24 months of receiving home-based epilepsy care (n = 97) PWE: people with epilepsy, HBC: home-based care, EQ-5D-3L: European Quality of Life Five-Dimension Three-Level

The EQ-5D-3L scale was used to assess the health status of people with epilepsy. Under the descriptive system, the following dimensions were assessed: mobility, self-care, usual activities, pain or discomfort, and anxiety or depression. These dimensions were assessed at two points in time: baseline (after recruitment) and after 24 months. Figure 2 depicts the percentage of PWE having some problems (level 2 + 3 of EQ-5D-3L scale) at baseline and after 24 months of receiving home-based epilepsy care. The bar in the diagram depicts the presence of some problems (level 2 + 3). The results show that after 24 months, there was a decrease in the percentage of PWE having some problems on all dimensions of the EQ-5D-3L scale (mobility, self-care, usual activities, pain or discomfort, and anxiety or depression).

**Figure 3:**
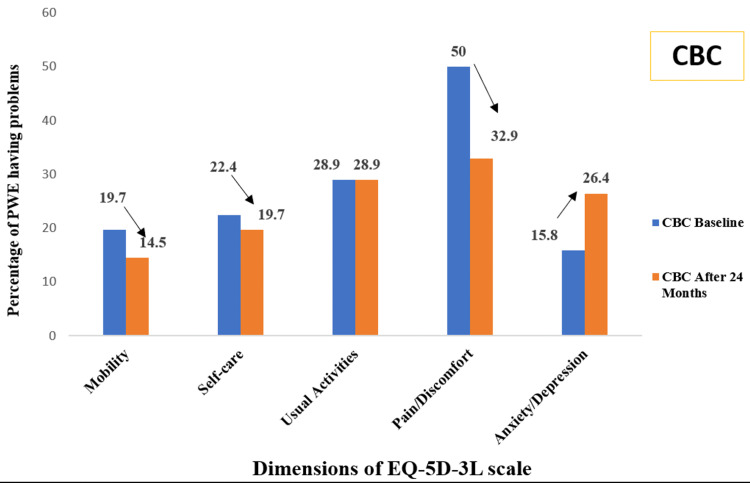
Percentage of PWE having some problems (level 2 + 3) at baseline and after 24 months of receiving clinic-based epilepsy care (n = 76) PWE: people with epilepsy, CBC: clinic-based care, EQ-5D-3L: European Quality of Life Five-Dimension Three-Level

Figure 3 shows the percentage of PWE having some problems (level 2 + 3) at baseline and after 24 months of receiving clinic-based epilepsy care. There was a decrease in the percentage of PWE having problems as per the dimensions mobility, self-care, and pain/discomfort; however, there was no change in the percentage of PWE having problems related to usual activities. Most importantly, the percentage of PWE having anxiety or depression increased after 24 months of care.

**Figure 4:**
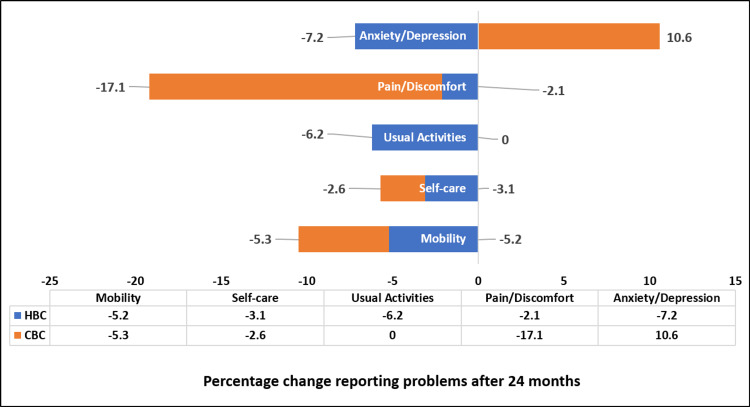
Percentage change in PWE reporting some problems (level 2 + 3) after receiving 24 months of epilepsy care as per various dimensions of the EQ-5D-3L scale (N = 173) HBC: home-based care, CBC: clinic-based care, PWE: people with epilepsy, EQ-5D-3L: European Quality of Life Five-Dimension Three-Level

Figure 4 shows the percentage change in problems after 24 months among PWE receiving home-based and clinic-based epilepsy care. There was a negative change in percentage for all dimensions, which means that there was a decrease in the percentage of PWE having problems, but the anxiety/depression dimension in the clinic-based epilepsy care group showed a positive change, i.e., there was an increase in anxiety or depression after 24 months. The possible reason could be that the PWE in home-based care were receiving education and counseling about self-management of epilepsy, social functioning, and stigma abrogation, while the clinic-based epilepsy care group was receiving routine care (although this is not under the study).

Calculation of index scores

The obtained health states were noted and converted into index scores as per the given value sets. The value sets for the health states in India were not available; therefore, Chinese value sets were used in the study [16]. The EuroQOL Foundation recommends selecting one of the nearby/similar country value set in the absence of a country-specific value set [15].

A score of “1” indicates perfect health. The higher the value (close to 1), the better the health. Both groups were similar as per the baseline index scores (p = 0.503). The mean EQ-5D-3L index scores for the HBC group at baseline were 0.88 ± 0.15, while after 24 months, they increased to 0.94 ± 0.17. The baseline mean index scores for the CBC group were 0.89 ± 0.21, while after 24 months, the value increased to 0.90 ± 0.19 (Table 3). The mean difference in index scores in the HBC group showed a higher difference than in the CBC group (0.06 ± 0.1 versus 0.01 ± 0.1). However, the difference found between the two groups was statistically not significant at p = 0.067.

**Table 3:** Comparison of the EQ-5D-3L index scores at baseline and after 24 months in PWE receiving home-based and routine clinic-based epilepsy care (N = 173) The closer the index value is to 1, the better the health; 1 means a perfect health state. EQ-5D-3L: European Quality of Life Five-Dimension Three-Level, PWE: people with epilepsy, HBC: home-based care, CBC: clinic-based care, SD: standard deviation, NS: not significant

Groups	EQ-5D-3L index scores		Mean difference	Z value	p value
	Baseline	After 24 months			
	Mean ± SD	Mean ± SD			
HBC (n = 97)	0.88 ± 0.15	0.94 ± 0.17	0.06 ± 0.1	-0.722	0.067^NS^
CBC (n = 76)	0.89 ± 0.21	0.90 ± 0.19	0.01 ± 0.1		
Z value	-0.669				
p value	0.503^NS^				

Assessment of the EQ visual analog scale (EQ-VAS)

Health was self-assessed using the EQ-VAS scale at two points in time: baseline and after 24 months. Both groups were similar as per baseline EQ-VAS scores at p = 0.439. Table 4 shows that people in both groups tended to improve their quality of life after receiving 24 months of care. However, the difference found between the two groups is statistically not significant as calculated using the Mann-Whitney test (Table 4).

**Table 4:** Comparison of VAS scores at baseline and after 24 months among PWE receiving home-based care and routine clinic-based epilepsy care (N = 173) VAS: visual analog scale, PWE: people with epilepsy, HBC: home-based care, CBC: clinic-based care, SD: standard deviation, minimum value: 0, maximum value: 100, NS: not significant

Groups	EQ-VAS score		Mean difference	Z value	p value
	Baseline	After 24 months			
	Mean ± SD	Mean ± SD			
HBC (n = 97)	67.1 ± 24.7	75.2 ± 27.6	8.1 ± 15.0	-1.605	0.10^NS^
CBC (n = 76)	64.1 ± 25.2	73.8 ± 25.4	9.7 ± 12.7		
Z value	-0.773				
p value	0.439^NS^				

Association of EQ-5D-3L scores with sociodemographic and clinical profile

The EQ-5D-3L index scores were significantly associated with education level (p = 0.003), working status (p = 0.001), age at seizure onset (p = 0.012), frequency of seizures (p = 0.001), presence of comorbidities (p = 0.002), and adverse drug reactions (p = 0.012). The EQ-VAS scores were significantly associated with education level (p = 0.012), working status (p = 0.001), age at seizure onset (p = 0.004), frequency of seizures (p = 0.001), treatment regimen (p = 0.005), presence of comorbidities (p = 0.005), and adverse drug reactions (p = 0.031).

## Discussion

The study aimed to assess the quality of life of people with epilepsy using a generic EQ-5D-3L scale. Most of the studies done previously to assess HRQOL in people with epilepsy measured HRQOL at a single point in time [7,12,14,17]. There is very limited literature on quality of life among PWE using the EQ-5D-3L scale, and this is the first study where two different types of epilepsy care (home-based epilepsy care and clinic-based epilepsy care) were compared for change in the quality of life.

In the present study, the five dimensions of the EQ-5D-3L scale, namely, mobility, self-care, usual activities, pain or discomfort, and anxiety or depression, were also compared for the percentage of problems present at baseline and after 24 months of epilepsy care. There was a decrease in the percentage of problems on all dimensions of the EQ-5D-3L scale among PWE receiving home-based epilepsy care. Among the PWE receiving clinic-based care, there was a decrease in the percentage of PWE having problems with mobility, self-care, and pain/discomfort. However, there was no change in the percentage of PWE having problems related to usual activities, and the percentage of PWE having anxiety or depression increased after 24 months of care. The possible reason could be that the PWE in home-based care were receiving education and counseling about self-management of epilepsy, social functioning, and stigma abrogation, while the clinic-based epilepsy care group was receiving routine care (although this is not under the study). Similar results were found in another study, where an improvement in all dimensions of the EQ-5D-3L scale was observed when compared to baseline values [14].

The mean EQ-5D-3L index scores for the HBC group at baseline were 0.88 ± 0.15, while after 24 months, they increased to 0.94 ± 0.17. The baseline mean index scores for the CBC group were 0.89 ± 0.21, while after 24 months, the value increased to 0.90 ± 0.19. The mean difference in index scores in the HBC group showed a higher difference than in the CBC group (0.06 ± 0.1 versus 0.01 ± 0.1). However, the difference found between the two groups was statistically not significant at p = 0.067. Similar results were found by de Souza et al. (2018), where the sample population tended toward a gain in quality of life after second follow-up visits to the outpatient clinic [13].

The EQ-5D-3L index scores and EQ-VAS scores were significantly associated with education level, working status, age at seizure onset, frequency of seizures, treatment regimen, presence of comorbidities, and adverse drug reactions. The variables found to be associated with poor HRQOL in people with epilepsy are similar to the factors found in other studies [7,17].

The study has some limitations, as the chosen cohorts were people with epilepsy who had previously been enrolled in a community-based randomized controlled trial. The generic instrument has been used to compare the burden of epilepsy in comparison to other disorders, but it does not give complete details about epilepsy. The number of subjects was limited in the groups, the survey location could also be expanded, and further studies can be replicated with larger cohorts to establish more evidence. The quality of life between children and adults could be compared separately to learn much about the differences between them.

## Conclusions

The results of the study emphasize that epilepsy has a negative impact on quality of life. There was an improvement in QOL from baseline after dedicated care in both groups. The results showed a higher QOL among the people in the HBC group as compared to the CBC group, but the difference was not statistically significant. The problems related to mobility, self-care, usual activities, pain/discomfort, and anxiety/depression have been significantly reduced in the HBC group. Having low levels of education, not having a job, starting to have seizures at a young age, having seizures more often, receiving more than antiepileptic drugs, and the presence of other health problems and side effects are factors found to be associated with poor QOL among people with epilepsy.
